# Limbal stem cell defficiency associated with primary adrenocortical insufficiency

**DOI:** 10.3205/oc000091

**Published:** 2019-02-06

**Authors:** Nurgül Örnek, Kemal Örnek, Tevfik Ogurel

**Affiliations:** 1 Department of Ophthalmology, School of Medicine, Kırıkkale University, Yahşihan, Turkey; 2 Department of Ophthalmology, Kudret Eye Hospital, Ankara, Turkey

**Keywords:** limbal stem cell deficiency, primary adrenocortical insufficiency

## Abstract

**Objective:** To report a female patient with bilateral limbal stem cell deficiency (LSCD) due to primary adrenocortical insufficiency (PAI).

**Methods:** Case report

**Results:** A 40-year-old female patient had blurry vision, foreign body sensation, tearing, and photophobia for several years. On examination, corneal epithelial haze, surface irregularity, and superficial neovascularization were observed. There was a dull and irregular reflex from the conjunctivalized corneal surface. Medical history revealed that she had a diagnosis of PAI for 11 years and received hormone replacement (fludrocortisone acetate) therapy. With the clinical presentation and examination, the diagnosis was compatible with LSCD. Frequent ocular lubricant and topical steroid drops were initially started and topical cyclosporine treatment was planned for the long term. After 3 weeks, there was no corneal superficial neovascularization and epithelial haze, peripheral stromal haze was still observed.

**Conclusion:** LSCD may rarely be associated with PAI. In patients with LSCD, systemic evaluation should be made to rule out PAI.

## Introduction

Limbal stem cells maintain the normal ocular surface homeostasis and the healing of corneal epithelial wounds and act as a barrier against the invasion of the cornea by conjunctival epithelial cells and vessels. Limbal stem cell deficiency (LSCD) is a pathologic condition that results from dysfunctional and/or an insufficient number of limbal stem cells [1]. All disorders causing LSCD have significant ocular surface inflammation in common except for some congenital conditions and neoplasia [2].

Primary adrenal insufficiency (PAI) is a life-threatening disease that results from destruction or dysfunction of 90% or more of both adrenal cortices. The most common cause of PAI is autoimmune destruction of the adrenal gland and autoimmune PAI may also occur as a component of several autoimmune polyendocrinopathy [3]. Association of LSCD and autoimmune polyendocrinopathy have been reported in previous studies [4], [5], [6], [7], [8]. 

As far as we know, there is no case of LSCD and PAI without any other coexisitng endocrinopathy in the current literature.

## Case description

A 40-year-old female applied to the outpatient clinic with blurry vision, foreign body sensation, tearing, and photophobia lasting for several years. Her medical history revealed that she had primary adrenocortical insufficiency for 11 years and received hormone replacement (fludrocortisone acetate) therapy. 

**Figure 1:**
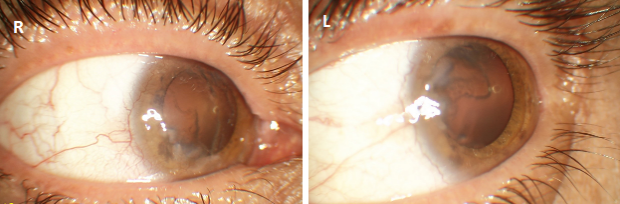
Corneal epithelial haze, classic “whorled” epithelium extending to the central cornea and superficial peripheral corneal neovascularization before treatment

Best corrected visual acuity was 5/10 in the right eye and 6/10 in the left eye. Slit-lamp examination revealed a dull and irregular reflex from the corneal surface. There was corneal epithelial haze and classic “waterfall” or “whorled” epithelium extending to the central cornea. Superficial peripheral corneal neovascularization was also observed in both eyes (Figure 1 (Fig. 1)). Serum cortisone level was lower than normal [31.62 nmol/L (normal range, 64–536)]. Serum parathormone, thyroid hormone, thyroid stimulating hormone, sodium, calcium, potassium, and phosphorus levels were within normal limits in various blood tests. With the history and clinical presentation, the diagnosis was compatible with partial LSCD. 

**Figure 2:**
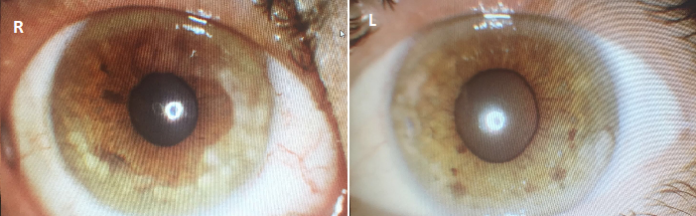
Stromal scar in peripheral cornea of both eyes after 3 weeks of treatment. There was no corneal neovascularization and corneal epithelial haze.

We started treatment with frequent ocular lubricant and topical steroid drops for ocular surface disease in the acute phase to control the inflammation and planned to go on with topical cyclosporin for the long term. The patient was already taking systemic steroid treatment with oral cortisone at the endocrinology department. After 3 weeks, there was no corneal neovascularization and corneal epithelial haze, but a stromal scar remained at the peripheral cornea of both eyes (Figure 2 (Fig. 2)).

## Discussion

Limbal stem cell deficiency may be complete or partial. In complete LSCD, no stem cells are available to repopulate the corneal surface, but in partial form some remaining corneal stem cell tissue is present [2]. In another classification, LSCD is categorized in 2 groups. In category 1, there is limbal stem cell destruction caused by chemical/thermal burns, Stevens-Johnson syndrome, multiple surgeries, cryotherapies, contact lens wear, and severe microbial keratitis. But in category 2, there is gradual loss of stem cell functions over time due to multiple endocrine deficiency, aniridia, neurotrophic keratopathy, peripheral inflammmatory keratopathy, limbitis, or idiopathic. Limbal stem cell transplantation and surface reconstruction are used in category 1 and modulation of hormonal, neuronal, vascular, inflammatory, and developmental factors are treatment alternatives in category 2 [8]. 

In the case we reported here, partial LSCD was present and reduced serum cortisone levels may be accused for partial LSCD. We started conservative topical treatment together with systemic hormone replacement and the treatment result was very satisfactory. 

Association of LSCD and multiple endocrine deficiency (MEN) has been reported in few previous studies [4], [5], [6], [7], [8]. In all those reported cases, in additon to primary adrenocortical insufficiency (Addison disease), there were also conditions like chronic mucocutaneous candiasis, idiopathic hypoparathyroidism, ectodermal dystrophy, and immune disorders, which made the diagnosis of MEN or a subgroup of MEN that is APECED syndrome (autoimmune polyendocrinopathy/candiasis/ectodermal dystrophy). 

This case is different from the previous ones as including only Addison disease without any other coexisting situations. It seems that inflammmation is the triggering factor of LSCD in these eyes and serum cortisone levels have critical importance in regulating the inflammation level the on ocular surface. Ocular surface inflammation may lead to an environment rich in inflammatory mediators and proapoptotic cytokines and it is more probable that limbal stem cells may be susceptible to cell death in such an environment. 

## Conclusion

LSCD without any underlying etiology should undergo a systemic workup including serum cortisone levels. In additon to frequent lubrication, anti-inflammatory treatment with topical corticosteroids in the acute symptomatic phase and topical cyclosporine treatment with systemic cortisone replacement therapy in the long term seem to have a higher success rate in patients with partial LSCD and PAI. 

## Notes

### Competing interests

The authors declare that they have no competing interests.
